# Corpus callosum agenesis and rehabilitative treatment

**DOI:** 10.1186/1824-7288-36-64

**Published:** 2010-09-17

**Authors:** Matteo Chiappedi, Maurizio Bejor

**Affiliations:** 1 Rehabilitation Unit, "Santa Maria alle Fonti" Medical Center, Don Carlo Gnocchi ONLUS Foundation, Salice Terme (PV), Italy; 2 Department of Neurological Sciences, University of Pavia, Pavia, Italy; 3 Francesco Bonaccorsi Foundation, Milan, Italy; 4 Department of Surgical, Resuscitative, Rehabilitative and Transplant Sciences, University of Pavia, Pavia, Italy

## Abstract

Corpus callosum agenesis is a relatively common brain malformation. It can be isolated or included in a complex alteration of brain (or sometimes even whole body) morphology. It has been associated with a number of neuropsychiatric disorders, from subtle neuropsychological deficits to Pervasive Developmental Disorders.

Etiology and pathogenetic mechanisms have been better understood in recent years, due to the availability of more adequate animal models and the relevant progresses in developmental neurosciences. These recent findings are reviewed (through a MedLine search including papers published in the last 5 years and most relevant previously published papers) in view of the potential impact on children's global functioning and on the possible rehabilitative treatment, with an emphasis on the possibility to exploit brain plasticity and on the use of the ICF-CY framework.

## Background

### Clinical presentation

Corpus Callosum Agenesis (CCA) is among the most common brain malformations observed in humans [1]. Its incidence varies as a function of both diagnostic techniques and sample populations: in the general population, its estimated prevalence is 3-7 per 1000 birth, while in children with developmental disabilities it is 2-3 per 100 [2-4]. It is often associated with other anomalies such as Chiari II malformation (also known as proper Chiari malformation) with abnormal development of cerebellar vermis and medulla oblongata, which tend to descend into the foramen magnum, usually accompanied by myelomeningocele, basilar type encephalocele and disorders of neural migration (which occurs concurrently in human brain development) such as schizencephaly, lissencephaly, pachygyria, marked neuronal heterotopias. Recent neuroradiological findings [5] suggest that CCA might lie along a dysgenetic spectrum, including all commissural anomalies as part of an overall cerebral dysgenesis. Abnormal sulcation is common and suggests more diffuse white matter dysgenesis in these foetuses [6], even if some authors do not consider this as an additional brain abnormality [7]. The isolated form of CCA is however listed in OMIM (217990) and ORPHAnet (ORPHA200).

Patients with CCA have a clinical syndrome which had originally been thought to be a consequence of hemispheres' disconnection. Recent studies, however, pointed out that patients with CCA have abnormal microstructure and reduced volume of the Ventral Cingulum Bundle, suggesting that abnormalities in intrahemispheric white matter tracts may be an important factor [8]. Another interesting recent finding is a reduction in number of Van Economo neurons, large spindle-shaped neurons localized to anterior cingulated cortex and fronto-insular cortex, in patients with CCA; this is considered another consequence of the genetic disruption that caused the agenesis [9].

Retrospective chart reviews and cross-sectional cohort studies report that 30 - 40% of cases have identifiable causes; however, up to 75% of cases with isolated complete CCA do not have an identified cause [10].

The possible genetical etiology of CCA has been studied both in mouse models [11] and in humans [12]. No single gene has been proved to be implied in all patients with CCA, given that the corpus callosum can be missing in many different disorders: this probably reflects complex underlying mechanisms [10]. Environmental factors are relevant as well: this is evidenced by the effect of ethanol on corpus callosum development, so that CCA is a relatively common feature of Fetal Alcohol Syndrome [13].

### Diagnostic issues

CCA is a disorder of midline prosencephalic development, together with agenesis of septum pellucidum, affecting the commissural plate.

Other disorders included in this group are septo-optic dysplasia (affecting the commissural but also the chiasmatic plates) and septo-optic-hypothalamic dysplasia (affecting the commissural, chiasmatic and hypothalamic plates).

In CCA the superomedial aspects of the lateral ventricles are deformed by the fibers of the cerebral hemispheres that were destined to cross in the corpus callosum and that, with agenesis, course instead longitudinally as the bundles of Probst. Crescentic lateral ventricles result from the impression of medial ventricular wall by these bundles. The other relevant neuroradiological sign is the evertion of the cingulated gyri which can be seen in coronal scans.

Ultrasonography can be helpful, even if MRI is thought to be far superior at least for partial agenesis. Morphologically, two types of CCA can be distinguished: in type 1 axons are present but unable to cross the midline, forming large aberrant fiber bundles (Probst bundles), while in the less frequent type 2, axons fail to form [1].

The most relevant sonographic sign in sagittal views is the superior displacement of the third ventricle, while parasagittal views show that the medial cortical sulci radiates superiorly instead of horizontally and the absence of the normally echogenic pericallosal sulcus. Moreover, coronal scans show absence of callosum and Probst longitudinal bundles indenting the dorsomedial aspect of lateral ventricles.

With partial agenesis, the posterior portion is nearly always affected, with the notable exception of the anterior involvement that occurs when partial agenesis is associated with the holoprosencephalies [14]. It is perhaps understandable that partial agenesis is determinated by disturbances occurring later during the maturational phase compared to complete agenesis.

In MRI, the four components of the corpus callosum are best viewed on sagittal imaging although its relationship to the cerebral hemispheres is best shown on coronal images. The corpus callosum is a densely packed white matter structure, with high signal on T1-weighted and low signal in T2-weighted images after the age of 24 months. Up until that age, MRI imaging showns that myelinization is more advanced in the posterior parts of the corpus callosum when compared to the anterior regions [15]. Diffusion Tensor Imaging and tractography have been used to study inter-hemispheres connectivity. Given however the difficulties in distinguishing a complete absence of the corpus callosum from a severe hypoplasia, the term "abnormal corpus callosum" has been proposed as a way to catch all observed abnormalities.

Prenatal diagnosis of complete callosal agenesis is feasible from the midtrimester onwards by expert sonography [16]. In the axial view, suspicious findings are absent cavum septi pellucid and teardrop configuration of the lateral ventricles with possible ventriculomegaly; the non-visualization of the corpus callosum at transfontanellar ultrasound in either the sagittal or coronal plane is diagnostic [17]. More subtle findings, such as hypoplasia and partial agenesis of the corpus callosum, may also be recognized antenatally [18]. Fetal MRI is worthy of recommendation in order to reinforce a difficult sonographic diagnosis and at the same time to exclude possible additional cerebral anomalies which may be overlooked at ultrasound but may affect the outcome considerably [19].

### Developmental origin

Prosencephalic development occurs by inductive interactions under the primary influence of prechordal mesoderm mainly during the second and third months of gestation. Since the major inductive relationship of concern is between the notochord-prechordal mesoderm and the forebrain and it occurs ventrally at the rostral end of the embryo the term "ventral induction" is used.

Development of the prosencephalon can be divided in three sequential events: prosencephalic formation (which is altered in aprosencephaly and atelencephaly), prosencephalic cleavage (altered in holoprosencephaly and holotelencephaly) and midline prosencephalic development.

Midline prosencephalic development occurs from the latter half of the second month through the third month, when three crucial thickenings or plates of tissue become apparent (commissural, chiasmatic and hypothalamic plates). The earliest components of the corpus callosum appear at approximately 9 weeks gestational age; by 12 weeks gestational age and independent corpus callosum is definable at the commissural plate. The latter is a structure derived from cellular material filling a sulcus which becomes evident in the dorsal portion of the lamina terminalis during the seventh gestational week. The commissural axons from the cortex are attracted to the midline mainly by chemoattractans of the phylogenetically conserved netrin family [20]. Commissural axons then cross the midline and project alongside it, without recrossing. An explanation for the failure to recross is that midline cells, in addition to expressing netrin proteins, also express the repellent protein Slit, which signals repulsion by activating the Roundabout (Robo) receptor. The growth cones can cross once because they do not initially express Robo protein on their surface, but upon crossing, with a mechanism that is still poorly understood, they upregulate Robo protein on their surface and therefore become responsibe to Slit, which prevents them from recrossing. Several other molecules, such as cell adhesion molecules, laminins, receptor protein tyrosine kinases, receptor protein tyrosine phospatases and semaphorins also have important roles as mediators of axon guidance [21]. Moreover the role of glial cells cannot be overlooked [22]. Bi-directional growth, beginning at the interface of the genu and body, leads to the development of the genu, followed by the body, the splenium and the rostrum; this process is completed by approximately 20 weeks of gestation [23,24]. Subsequent thickening of this structure occurs as a result of growth of crossing fibers during organizational events [25]. In the last 30 years the understanding of callosal development has increased because transgenic mouse models became available and studied [26].

Another important research line is the one considering chromosomal rearrangements with CAA as a possible feature. The number of patients with CAA in which chromosomal rearrangements are found has increased following technical improvements (i.e. from conventional cariotyping to subtelomeric and array-CGH analysis) [27]. Candidate genes have been located expecially on chromosome 1 [28-30], but also on 3, 7, 8, 13, 15, 18, 21 [31].

A recent attempt to classify all midbrain-hindbrain malformations has been proposed by Barkovich, Millen and Dobyns [31]. According to a comprehensive review of embryological and genetic findings, these authors have proposed four main categories:

• malformations secondary to early anteroposterior and dorsoventral patterning defects or to misspecification of midbrain or hindbrain germinal zones;

• malformations associated with later generalized developmental disorders that significantly affect the brainstem and cerebellum;

• localized brain malformations that significantly affect the brain stem and the cerebellum;

• combined hypoplasia and atrophy of putative prenatal onset degenerative disorders.

This classification could explain the developmental origins of malformations associated to CAA and the fact that an abnormal corpus callosum is found in many severe disorders of midbrain - hindbrain development [27]. A list of the most commonly syndromes associated with CCA is shown in Table 1.

**Table 1:** Some complex genetic syndromes with CCA as a possible feature

Syndrome	Gene (chromosomal region)	OMIM #
*Autosomal dominant*		

Apert syndrome	FGFR2 (10q26)	101200

Basal cell nevus syndrome	PTCH (9q22.3)	109400

Miller-Dieker syndrome	LIS1 (17p13.3)	247200

Mowat-Wilson syndrome	ZFHX1B (2q22)	235730

Opitz GBBB syndrome	not defined (22q11.2)	145410

Rubinstein-Taybi syndrome	CREBBP (16p13.3) EP300 (22q13)	180849

*Autosomal recessive*		

Acrocallosal syndrome	GLI3 (7p13)	200990

Andermann syndrome	SLC12A6 (15q13-q14)	218000

DeMorsier syndrome (septo-optic dysplasia)	HESX1 (3p21.2-p21.1)	182230

Fukuyama syndrome (congenital muscular dystrophy)	FCMD (9q31)	253800

Joubert syndrome	AHI1 (6q23.2-q23.3)	608629

Meckel-Gruber syndrome	not defined (17q22-q23)	249000

Muscle-Eye-Brain disease	POMGNT1 (1p34-p33)	253280

Walker-Warburg syndrome	FCMD (9q31)	236670

*X-linked*		

Aicardi syndrome	not defined (Xp22)	301040

FG syndrome	not defined (Xq12-q21.31)	305450

Opitz GBBB syndrome	MID1 (Xp22)	300000

X-linked lissencephaly	DCX (Xq22.3-q23)	300067

## Rehabilitation

The brain's complexity arises from its connectivity, as is highlighted by the disproportionate increase in white matter volume throughout primate evolution [32]. Since the corpus callosum, with over 190 million axons, is the largest structure connecting the two cerebral hemispheres [33], its importance is self-evident. Although there has been debate about whether the connections are primarily excitatory (integrating information across hemispheres) or inhibitory (allowing the hemispheres to inhibit each other to maximize independent functions), they appear to be primarily excitatory [34] with the secondary result to allow brain asymmetries and therefore independent functions [35].

Since there is a great variability in clinical outcome, different theories have been suggested. Interhemispheric connections could be re-routed through the anterior commissure [36] or other structures could be important, such as the recently discovered sigmoid bundle (which is a long heterotopic commissural tract, connecting the left frontal lobe with the right occipitoparietal cortex, seen in partial CCA) [37]. The long-known notion that CCA has a better outcome when is not associated with other brain malformations has been confirmed in recent studies [38]. On the other hand, the comparison between complete and partial CCA has revealed conflicting data, with multiple studies showing no difference in behavioural and medical outcomes between the two conditions, whereas one earlier study reported a worse outcome for individuals with complete CCA [39,18,12].

Since the percentage of completely "normal" patients is decreasing in more recent studies, one could hypothesize that a pattern of deficits in high-order cognition and social skills has become apparent as more individuals with primary CCA but without obvious neurological deficits have been identified and assessed with sensitive standardized neuropsychological measures [10]. A long follow up is therefore required even in apparently "benign" clinical conditions [40].

In general terms, three clinical patterns seem to be relatively common:

1) severe neuropsychiatric deficit, usually seen in complex brain malformative diseases in which CCA is only one feature (and often not the most relevant one, in terms of consequent disability);

2) other neurodevelopmental diseases, including autism [41], without a well defined role of CCA in the etiology of the disorder;

3) apparently benign conditions, with IQ in the normal range but relevant neuropsychological deficits. These ones include impairment of abstract reasoning [42], problem solving [43], comprehension of syntactic and linguistic pragmatics [44,45,42], and category fluency [42]. Parents frequently report health concerns such as feeding or sleep issues, elimination problems and unusual tolerance for pain [46,47]. Behavioural and emotional factors are frequently associated: tendency for deficient social cognition in individuals with ACC seems to stem from a combination of difficulty integrating information from multiple sources (e.g. verbal and visual ones), using paralinguistic cues for emotion, and understanding nonliteral speech [48]. This has been hypothesized to be the consequence of largely intact right hemisphere mechanisms supporting psychophysiological emotional response without having the possibility to integrate the intact mechanisms of the left hemisphere, due to the lack of communication and perhaps to a dysfunction of the anterior cingulated cortex [49]. This problems with emotional arousal can be the cognitive substrate for the psychopathological feature known as "alexitimia" (i.e. "lack of words to describe emotions"). A conduct disordered behaviour can stem from the inability to appropriately respond to complex demands, particularly under conditions of high stimulation, in a way somewhat similar to what happens in Rourke's Non Verbal Learning Disorder [50].

## Options for rehabilitation

One could wonder why rehabilitation units should challenge a disorder which originates from a more or less specific morphological alteration of the brain. There are many good reasons for doing that, both general and specific to CCA.

The general reason lies into the concept of CNS plasticity. It is well known that life events (obviously including rehabilitative treatments) can change the way our brain works and even its shape, if they have appropriate intensity and time length. This is particularly true for developing brains, but a place for CNS plasticity is now recognized even for adults and aged people.

The most important specific reason is the evidence that experience can modify established functional patterns. This has been shown to be true for instance for intermanual interactions [51]: rehabilitative treatment can be seen as an occasion to offer an experience which might in the long run modify neurological pathways through CNS plasticity. Studying sound lateralization in subjects with callosotomy, callosal agenesis or hemispherectomy, some authors have suggested that "congenital absence of the corpus callosum may result in processes of neural plasticity that compensate for the reduced transfer of auditory spatial information between the cortical hemispheres" [52]. It should also be considered that the corpus callosum reaches a size comparable to the adult one by 2 years of age, but it is one of the last systems to complete myelination, a process which starts at the fourth month of pregnancy but continues into adulthood [53].

An important rehabilitative target is therefore the possibility to exploit CNS plasticity: even if an important structure such as the corpus callosum is lacking, we can offer a specific exercise and a modified environment to improve functional and structural brain adaptation. In this way a spontaneous compensation through CNS plasticity can be enhanced, so that more complex stimuli and situations can be correctly processed and/or processing time can be reduced without losing accuracy. This rehabilitative target can be obtained through a specific training and using an adequate setting.

Other interesting studies have shown that the ability of patients with CCA to compensate their deficits depends on the task constraints: this has been proven for instance studying the ability to precisely time events in coordinate bimanual actions [54]. CNS plasticity is likely to be insufficient to compensate any neuropsychological deficit in any possible situation, as for instance suggested for synchronization of multimodal lateralized information [55] or for perceptual priming [56]. This implies that another relevant rehabilitative goal is the possibility to detect those situation where deficits can lead to a significant impairment and to activate helpful strategies (e.g. asking for explanations when a metaphor is used, given the high possibility to misunderstand its meaning). Interestingly, it is possible that this could lead to an improvement of the neuropsychological functioning, activating brain plasticity that leads to a non-standard use of cerebral areas (as in the case of language [57]).

Parents of individuals with CCA and relatively normal neuropsychiatric conditions consistently and reliably describe impaired social skills and poor personal insight as the features that interfere most prominently with the daily lives of their children [58]. Specific traits include emotional immaturity, lack of introspection, impaired social competence, general deficits in social judgement and planning, and poor communication of emotions [59,60]. Consequently, these patients often have impoverished and superficial relationships, suffer social isolation and have interpersonal conflict both at home and at school due to misinterpretation of social clues.

This implies that parents and teachers should be offered a counselling to help them finding ways to approach and help these children, without being stopped by their neuropsychological deficits but trying to overcome them with adequate strategies.

## Conclusions

Some suggestions can be drawn from current scientific literature concerning CCA.

First, the treatment should be started as soon as possible, in order to prevent secondary complications (e.g. social exclusion due to the above described difficulties) and to exploit CNS plasticity as much as possible.

Patients with severe neuropsychiatric disorders (developmental delay, autistic features, mental retardation) tend to present relevant problems quite early (usually by the age of 3 or 4 years). However, patients should be monitored at least up to school age, to detect more subtle neuropsychological deficits (which can however be significant for the patient and his/her family).

It is worth underlying that the functional diagnosis, evaluating in detail patient's points of weakness and strength as well as the real possibilities of the environment to support him, should guide the choice among these treatment options and the possible goals to look for. In other words, one should bear in mind that the aim of rehabilitation is improving patient's global functioning, not just treating a single and specific part of his body (such as the corpus callosum) or a single function (such as inter-hemispheric connectivity in CAA).

The model proposed in the International Classification of Functioning, Disability and Health [61] can be useful as a frame to guide the rehabilitative treatment. The recently published Children and Youth version of the ICF [62] maintains the focus on the dynamic interaction between the subject and the environment (bio-psycho-social model).

The subject's functioning can be described in terms of body structures (eventually altered, as in CCA) and functions (i.e. physiological functions performed by one or more structures), but also in terms of activities (i.e. actions the subject can or cannot perform) and participation (defined as the possibility to be involved in life situations). Activities and participation can be assessed as a performance (i.e. what the subject can or cannot do in his actual living environment) or as capacity (i.e. what the subject can or cannot do without any external intervention). The environment can act as facilitators (if they tend to increase subject's participation) or as barriers (if they tend to decrease subject's participation).

This model implicitly predicts that rehabilitative results can be much different, as they are the final result of a combination of many factors, including the extension of brain malformation and functional alteration, the possibility to offer a prompt and adequate treatment and parent's and teacher's possibility to adapt their behaviours to favour child's development. It can guide the interpretation of the assessment, because it allows to identify problems and use the right kind of intervention: this is especially important for situations such as CAA, which is present (either as an isolated alteration or as part of a complex malformative picture) in a wide range of clinical disorders. This also implies that any rehabilitative treatment has to be individually tailored, using different interventions according to the specific needs and possibilities of every single patient.

In few cases, devices of different kind can help the child to overcome specific limitations: their use should however be reserved to those situation where the possibility to enhance child's functioning is too low. Possible rehabilitative interventions can include:

• speech therapy: to improve child's language and reading/writing skills; different techniques are used, according to the specific deficits and skills of the subject;

• physiotherapy: mainly to reduce motor problems and sequelae in complex neurodevelopmental disorders;

• psychomotor therapy: to improve child's development in a general meaning, using a mixture of motor, cognitive and relational training. It combines different play-based techniques to improve the knowledge of oneself and of the world, through motor functioning, with a consequent ability to establish a relationship with the human and non-human environment [63];

• occupational or educational therapy: used in older children, with both a one-to-one or small groups approach; they tend to increase general social and cognitive abilities using "goal directed" exercises;

• psychotherapy: usually for children with a higher functional level. In current indexed literature, cognitive and behavioural approaches have been more often used, so that the possible role of other psychotherapeutic approaches (e.g. psychodynamic or inter-personal) is not clearly established,

• parent training: tries to improve parental attitude towards their child, helping them to accept his limitations and to favour as much as possible his global development;

• counselling for teachers: to provide the better adapted learning opportunities.

Whatever the chosen intervention(s), the patient should be monitored to adapt rehabilitative treatments to his (and often to his family's) changing needs.

## Competing interests

The authors declare that they have no competing interests.

## Authors' contributions

Both authors equally contributed to this paper. Both authors have read and approved the final manuscript.
